# Comparison of real time and malachite-green based loop-mediated isothermal amplification assays for the detection of *Plasmodium vivax* and *P*. *falciparum*

**DOI:** 10.1371/journal.pone.0234263

**Published:** 2020-06-11

**Authors:** Keare A. Barazorda, Carola J. Salas, Danett K. Bishop, Naomi Lucchi, Hugo O. Valdivia

**Affiliations:** 1 Department of Parasitology, U.S. Naval Medical Research Unit 6 (NAMRU-6), Lima, Peru; 2 NGO Prisma, Lima, Peru; 3 Division of Parasitic Diseases and Malaria, Malaria Branch, Center for Global Health, Centers for Disease Control and Prevention, Atlanta, Georgia, United States of America; Instituto Rene Rachou, BRAZIL

## Abstract

The current context of malaria elimination requires urgent development and implementation of highly sensitive and specific methods for prompt detection and treatment of malaria parasites. Such methods should overcome current delays in diagnosis, allow the detection of low-density infections and address the difficulties in accessing remote endemic communities. In this study, we assessed the performance of the RealAmp and malachite-green loop mediated isothermal amplification (MG-LAMP) methodologies, using microscopy and conventional nested-PCR as reference techniques. Both LAMP techniques were performed for Plasmodium genus, *P*. *falciparum*, and *P*. *vivax* identification using 136 whole blood samples collected from three communities located in the Peruvian Amazon basin. Turnaround time and costs of performing the LAMP assays were estimated and compared to that of microscopy and nested-PCR. Using nested-PCR as reference standard, we calculated the sensitivity, specificity and 95% confidence interval (CI) for all methods. RealAmp had a sensitivity of 92% (95% CI: 85–96.5%) and specificity of 100% (95% CI: 89.1–100%) for species detection; sensitivity and specificity of MG-LAMP were 94% (95% CI: 87.5–97.8%) and 100% (89.1–100%), respectively. Whereas microscopy showed 88.1% sensitivity (95% CI: 80.2–93.7%) and 100% specificity (95%: 89.1–100%). The turnaround time and costs of performing the LAMP assays were lower compared to those associated with nested-PCR but higher than those associated with microscopy. The two LAMP assays were shown to be more sensitive and simple to implement than microscopy. Both LAMP methodologies could be used as large-scale screening tests, but the MG-LAMP assay uses a simple, portable heat-block while the RealAmp requires a RealAmp machine or a real-time PCR machine. This makes the MG-LAMP an appropriate choice for malaria surveillance studies in endemic sites. Use of LAMP tests in active case detection of *Plasmodium* parasites could help to detect positive malaria cases early.

## Introduction

Malaria remains an important health problem in the Americas with an estimated 753,700 cases in 2018[1]. The majority of mortality due to malaria in this region is caused by *Plasmodium falciparum*. However, *P*. *vivax* is responsible for nearly 79.5% of all reported cases [1]. *P*. *vivax* infections are commonly asymptomatic and submicroscopic and consequently difficult to diagnose and treat [2, 3]. Therefore, these untreated *P*. *vivax* infections are responsible for maintaining transmission in endemic areas [4].

Currently, malaria identification and case management relies on confirmation of positive cases by microscopy or rapid diagnostic tests (RDTs), typically HRP2-based RDTs [5]. One of the challenges for malaria surveillance and control programs is the timely identification of low-density infections not detected by the routine microscopy and standard RDTs. Both methodologies present limitations that impair accurate parasite detection. The limitations of microscopy include the need for highly skilled personnel, the long turnaround time and relatively high operational sensitivity of approximately 50 parasites/μL. The use of RDTs in the Americas is limited due to the higher false negative rates [6] caused by the existence of parasites that have deleted the HRP2 and HRP3 genes [7]. Given these limitations and the generally low sensitivity of non-HRP2 based malaria RDTs, there is a critical need to develop alternative means for accurate diagnosis of malaria cases [6].

Molecular based approaches like Polymerase Chain Reaction (PCR) are highly sensitive and specific methods that allow accurate identification of the infecting species. In 2014, during the WHO Evidence Review Group on Malaria Diagnosis in low transmission settings, it was deemed that a molecular test must be able to detect two or fewer parasites per microliter (2 parasites /μL) to be a ‘significant improvement’ over expert microscopy. Many malaria molecular tests have limits of detection of 2 or fewer parasites /μL, however, their routine use is limited due to technical challenges and the requirements for highly sophisticated equipment and laboratory capacity [8]. Some of the challenges to using PCR-based molecular methods have been overcome by the use of simpler nucleic acid-based tests such as the loop mediated isothermal amplification (LAMP) assays, reviewed in [9].

In contrast to standard PCR, LAMP is performed at a constant temperature between 60–65°C and does not require a heat denaturation step to start the amplification. In addition, LAMP uses a set of four to six primers, which increase the specificity of the reaction. DNA amplification during the LAMP results in turbidity due to the formation of magnesium pyrophosphate by product that has a direct correlation with the amount of DNA amplified and that can be visually observed [10]. However, relying on turbidity as a readout has been shown to be subjective and colorimetric LAMP assays [11–14] and the use of fluorescence dyes as readout of LAMP assays have been developed to overcome this subjectivity [15]. These characteristics makes LAMP a suitable choice for use in the screening of large numbers of samples in a short time and in low resources settings, which is key in the context of malaria surveillance and control [16].

Several malaria LAMP-based assays have been described to date [9]. Many of these have excellent diagnostic performances, detecting as few as 1 parasite/μl (*Illumi*gene LAMP), or 1–5 parasites/μl for the Loopamp MALARIA kit (EIKEN Chemical Co) and utilize a variety of read outs. The malachite green loop-mediated isothermal amplification (MG-LAMP) is a colorimetric assay that does not require any special read-out equipment except a small portable heat block and mini-centrifuge. It has a testing capacity of 38-samples per run and has been described for the detection of Plasmodium infection and *P*. *falciparum* and *P*. *vivax* [15, 17]. The RealAmp is a real-time LAMP based assay that uses a simple and portable device capable of performing both the amplification and detection (by fluorescence) of LAMP in one platform [12, 18, 19]. In this study, we report on the performance of the RealAmp and MG-LAMP assays to diagnose malaria compared to microscopy and nested–PCR.

## Materials and methods

### Study sites, sample selection and ethics

The region of Loreto is located in the northwest Peruvian Amazon basin and has an equatorial climate. Annual temperatures range between 24 to 33°C with annual precipitations from 9 to 12 meters. Loreto contributes with the vast majority of malaria cases reported in the country with 53,163 cases in 2017 [20]. The majority of malaria cases are caused by *P*. *vivax* in a ratio of 4 to 1 in relation to *P*. *falciparum*. In addition, malaria transmission is perennial in this region with seasonal increases of cases [21].

**Fig 1:**
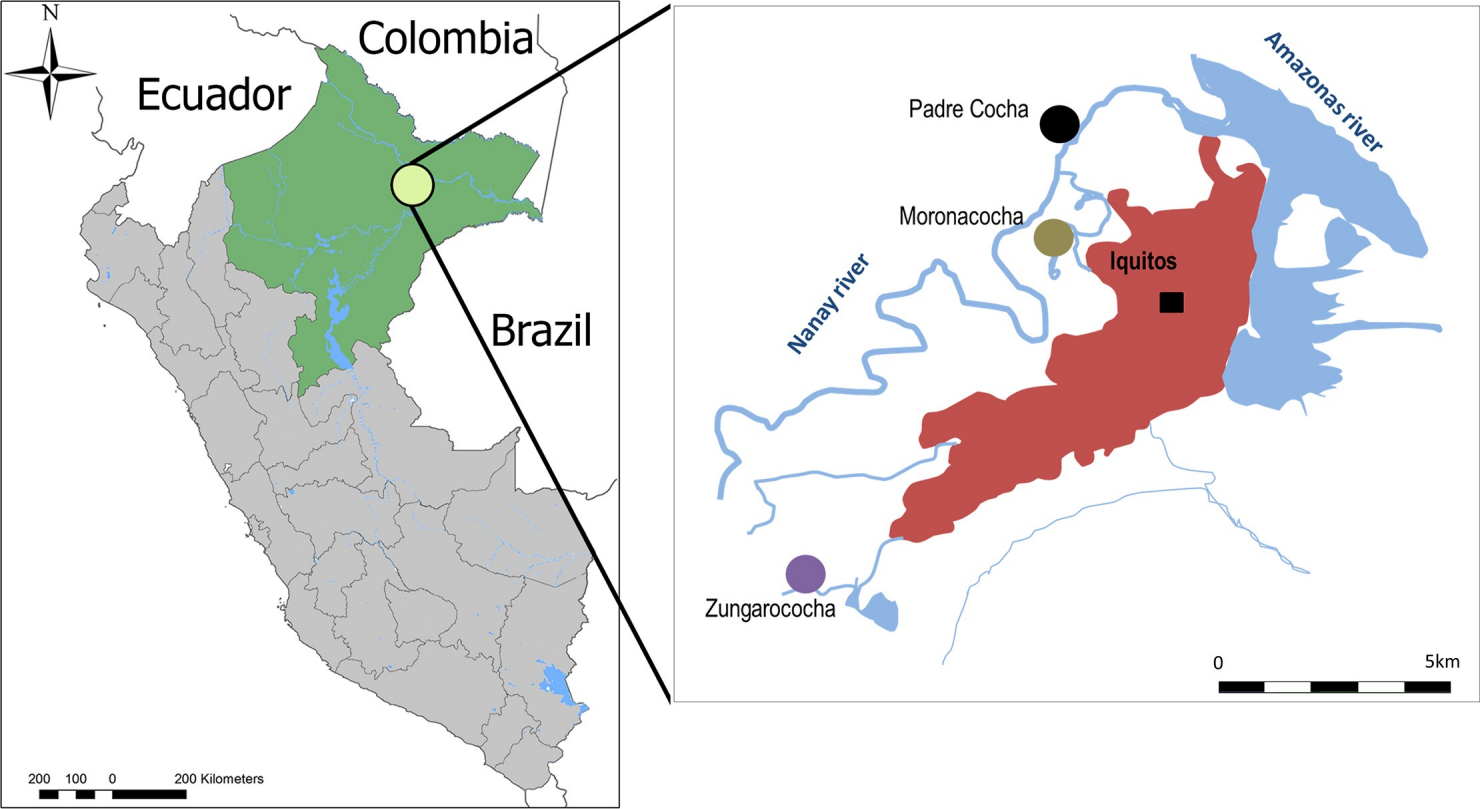
Map of the study sites located in the region of Loreto (green). The insert shows the location of the three participating communities depicted with circles and the city of Iquitos in red.

The clinical samples for this study were randomly selected from our database from two passive surveillance studies carried out between 2015 and 2016 in the department of Loreto, Peru (**Fig 1**). Both studies were approved by the Institutional Review Board of the U.S Naval Medical Research Unit 6 (NAMRU-6) in compliance with all applicable federal regulations governing the protection of human subjects (NMRCD.2007.0004 / NAMRU.2014.0031). All samples processed for this study had future use consent.

The first set of samples (106 participants) were from a cross-sectional study in the riverine communities of Padrecocha, Moronacocha and the city of Iquitos. Participants were recruited at health centers in these communities. Inclusion criteria for this study were age greater than 1 year with presence of fever or history of fever during the previous 72 hours and consent (and assent where appropriate).

The second set of samples (30 participants) came from a cohort study in Zungarococha, Iquitos. Participants for this study were recruited at their households. The inclusion criteria included age greater than 2 years old regardless of malaria symptoms, with consent (and assent where appropriate).

### Microscopy

Thick and thin blood smears were prepared and read at NAMRU-6 laboratory in Iquitos. Blood smears were stained with 10% Giemsa for 10 minutes following a standard operating procedure. A negative thick smear was one that lacked sexual or asexual parasites in 200 high power fields. Parasite densities were calculated counting the number of asexual parasites per at least 200 White Blood Cells (WBC) and the assumed 6000 WBC per microliter of blood. All slides were read by two independent microscopists; a third microscopist was engaged to sort discrepancies between the initial two microscopists.

### DNA extraction

All samples collected in Iquitos were transferred to NAMRU-6 Lima and kept at -80°C. DNA extraction was performed in early 2017 using 200μL of whole blood with the DNeasy Blood & Tissue kit (Qiagen)™ according to the manufacturer’s instructions and the resulting DNA was eluted in 70μL of elution buffer and stored at -20°C until use.

### 18srRNA-nested PCR

A nested-PCR targeting the small 18S rRNA subunit ribosomal RNA (ssrRNA) gene was conducted as previously described [22]. Both PCR reactions were carried out using 5 μl DNA template in a 50μl volume containing 1X buffer, 2mM MgCL_2_, 125μM dNTPs, 250nM of each primer and 1 unit of Taq Polymerase (Invitrogen, Waltham, MA). The PCR amplification products were analyzed in a 2% agarose gel. Nested- PCR was used as the reference test for all comparisons.

### RealAmp assay

The RealAmp assay was performed at the NAMRU-6 laboratory in Lima following a previously reported method with some modifications[12]. Briefly, all reactions were carried out in a volume of 12.5 μl that contained 2X in-house reaction buffer (40mM Tris-HCl pH8.8, 20mM KCl, 16mM MgSO_4_, 20mM (NH_4_)_2_SO_4_, 0.2% Tween -20, 1.6 M Betaine, 2mM of dNTPs each), 0.25μL of 1:400 SYTO 9 dye, 8 units of *Bst* Polymerase (New England Biolabs, Ipswich, MA) and 2μL of template DNA. Genus and species-specific primers for *P*. *vivax* and *P*. *falciparum* were used for the amplification of the 18S ribosomal RNA. The RealAmp assay was performed on an Mx3005P qPCR system (Agilent technologies, Santa Clara, CA) with fluorescence measured at 1 minute intervals under the FAM fluorescence channel (λ 494nm absorption/ 518nm emission). The genus and *P*. *falciparum* reactions were performed at 63°C whereas the *P*. *vivax* reaction was run at 64°C. All samples were run in triplicates and amplification was performed for 1 hour.

### Malachite green LAMP assay

The MG-LAMP was performed at the NAMRU-6 laboratory in Lima in a 20μL reaction volume, using the same components of the 2X reaction buffer as the RealAmp with the addition of 0.004% Malachite Green dye (MG), 8 units of *Bst* polymerase and 5μL of template DNA as previously described [15]. The amplification reaction was performed at 63°C for 60 minutes using a mini heat block (Gene Mate, Bio Express, Utah, US). The results were visually read by three independent technicians after 15 minutes post LAMP amplification. Positive samples were those defined by a green/blue color of malachite green, whereas negative samples remained colorless. All samples were processed first by the genus-screening assay and then by the species-specific assays for *P*. *falciparum* and *P*. *vivax*.

### Limit of detection of MG-LAMP and RealAmp assays

To assess the limits of detection (LoD) of the LAMP assays, samples with known parasite densities were prepared and tested in triplicate. For *P*. *falciparum*, we harvested the *P*. *falciparum* 7G8 reference strain following methods described by WWARN (http://www.wwarn.org). Briefly, parasites were cultured using (RPMI 1640, 0.025M HEPES, 0.02mg/mL Gentamicin, 0.20mM Hypoxanthine and 5%Albumax) until reaching a 5% ring stage parasitemia. Then, parasites were diluted tenfold from 100,000 parasites/μL to 1 parasite/μL with two-fold dilutions from 10 parasites/μL to 1.25 parasites/μL. This same method was used to determine the LoD for *P*. *vivax* using a field sample.

### Processing time and cost analysis

We estimated the turnaround time and costs of microscopy, nested-PCR, RealAmp and MG-LAMP for the specific identification of *Plasmodium*. Estimated cost included cost for reagents, materials for column-based DNA extraction and performing the PCR and does not include labor costs. Turnaround time was calculated as the time required from samples processing to getting results (include DNA extraction, master mix preparation, PCR reaction, and gel electrophoresis (if applicable).

### Data analysis

Sensitivity and specificity with 95% confidence intervals (95% CI) were calculated for microscopy, RealAmp and MG-LAMP using the nested-PCR results as the reference test. The agreement between diagnostic test was calculated using Kappa coefficient. Statistical differences between RealAmp and MG-LAMP were determined using the McNemar test. RealAmp and MG-LAMP assays were performed blinded to the results of PCR and microscopy. Data was analyzed using Stata, version, 13 (StataCorp LP, College station, TX)

## Results

### Analytical limit of detection of LAMP assays

**Fig 2:**
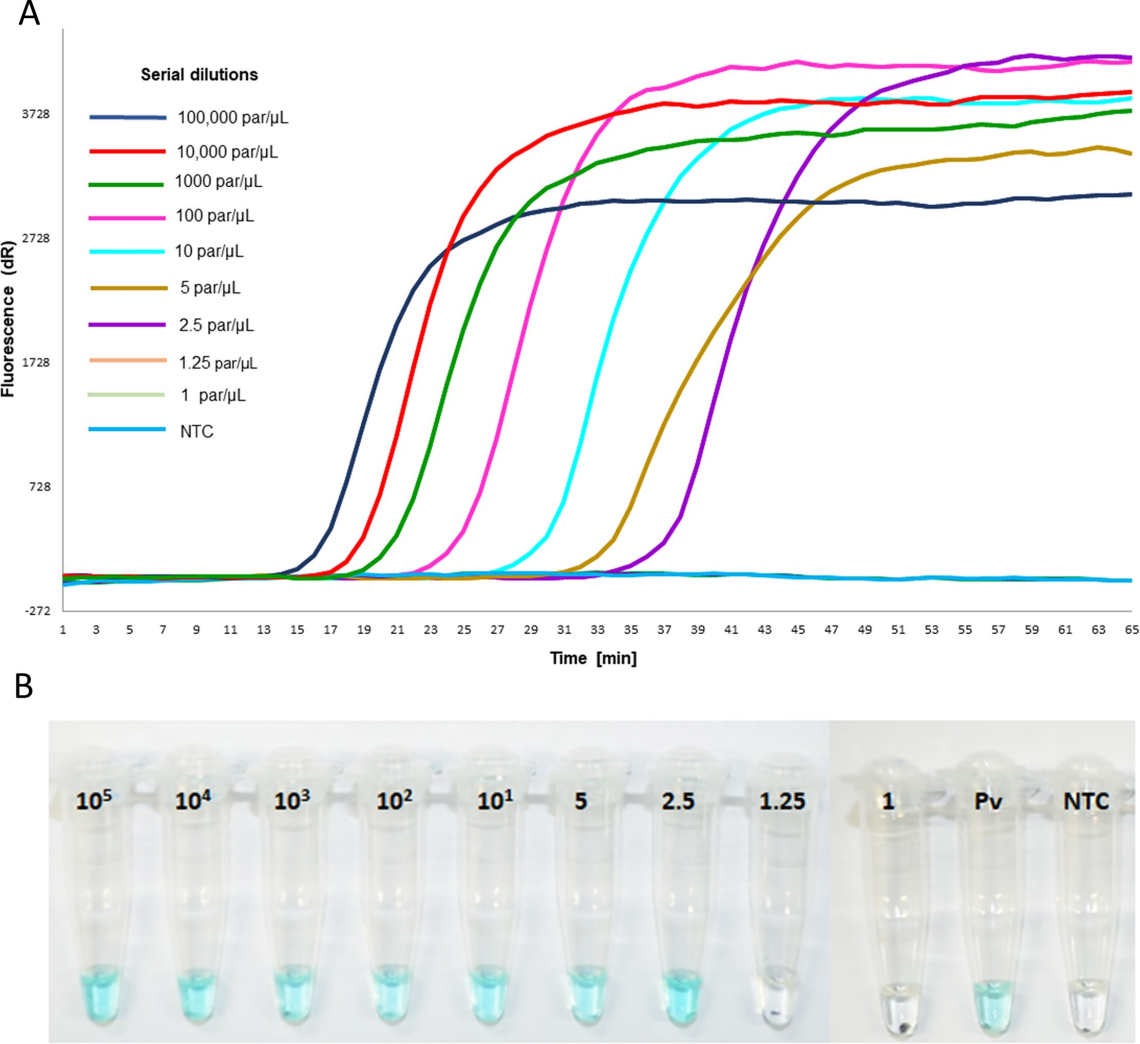
Limit of detection (LoD) of the genus-specific LAMP assays from 10-fold and 2-fold serial dilutions of *P*. *falciparum* reference strain 3D7. A. RealAmp amplification curves (positive) were observed from concentrations between 100,000(10^5^) and 2.5 parasites/μl dilution. B. Colorimetric MG-LAMP results can be visualized with the naked-eye, blue/green indicate positive whereas colorless negative. Pv: *P*. *vivax* control and NTC: no template control. Range 10^5^–1 = #parasites/μl.

To evaluate the LoD for RealAmp and MG-LAMP the reactions were performed in triplicates. The LoD for both the genus RealAmp and MG-LAMP was 2.5 parasites/μL (**Fig 2**). In addition, the LoDs for both assays for *P*. *falciparum* and *P*. *vivax* were 5 and 10 parasites/μL, respectively.

The genus RealAmp amplification time ranged from 16 to 38 minutes for the dilution range of 100,000 (10^5^) to 2.5 parasites/μL, respectively. In the case of MG-LAMP, there was 100% concordance by three independent readers in all positive parasite dilutions up to the described LoDs.

### Malaria tests results for field samples

A total of 136 samples were analyzed in this study. The average age of the participants is 29 years, regarding the sex: 57% were male (n = 78) and 43% female (n = 58). Using microscopy 34 samples were read as negative and 102 were read as positives (43 *P*. *falciparum*, 58 *P*. *vivax* and 1 mixed infection (*P*. *falciparum* and *P*. *vivax*)) with a median asexual parasitemia of 6,092.7 parasites/uL (range: 36–69,235 parasites/μL) and median gametocytemia of 1,167.8 parasites/uL (range: 12–44,490 parasites/μL). Nested-PCR identified a higher number of mixed infections resulting in 33 *P*. *falciparum*, 58 *P*.*vivax* and 11 mixed infections, Table 1.

**Table 1:** Summary of results performed by each methodology.

Species	Microscopy	Nested PCR	RealAmp	MG-LAMP
All positive (*Plasmodium* sp)	102	102	101	102
*P*. *falciparum*	43	33	40	40
*P*. *vivax*	58	58	57	58
**Mixed infection (Pf/Pv)**	1	11	4	4
**Negative**	34	34	35	34

Of the 102 positive samples, nested-PCR detected 11 (Pf/Pv) mixed infections, microscopy detected one whereas the RealAmp and MG- LAMP detected 4 (Table 2).

**Table 2:** Comparison of results of mixed infections (Pf/Pv) performed by each methodology.

Sample *	Microscopy	RealAmp	MG-LAMP
**1,2,5,6,7,9**	*P*.*falciparum*	*P*.*falciparum*	*P*.*falciparum*
**3**	Pf/Pv	Pf/Pv	Pf/Pv
**4,8**	*P*.*falciparum*	Pf/Pv	Pf/Pv
**10**	*P*.*falciparum*	Pf/Pv	*P*.*falciparum*
**11**	*P*.*falciparum*	*P*.*falciparum*	*Pf/Pv*

*Mixed infection (Pf/Pv) by nested-PCR.

Using nested-PCR as reference, microscopy had a sensitivity of 88.2% (95% CI: 80–93.5%) and specificity of 100% (95%: 87.4–100%). The genus-RealAmp method had 99.0% sensitivity (95% CI: 93.9–99.9%) and specificity 100% (95% CI: 87.4–100%) with a median amplification time of 20 minutes (range: 17–26 min) whereas the genus MG-LAMP had 100% sensitivity (95% CI: 95.5–100%) and 100% specificity (95% CI: 87.4–100%).

The species-specific RealAmp had a sensitivity of 92% (95% CI: 84.7–96.3%) and a specificity of 100% (95% CI: 87.4–100%). The sensitivity of the RealAmp was lower because 7 mixed infections and one P. vivax sample were not accurately detected by this assay (Table 1). The species-specific MG-LAMP had a sensitivity of 93% (95% CI: 85.9–97%) and a specificity of 100% (87.4–100%). The species-specific MG-LAMP classified 7 mixed infections as *P*. *falciparum* only infections (Table 2).

We calculated the kappa coefficient for all the methods using nested-PCR as reference test. Our results show that microscopy had 91,2% agreement (kappa = 0.79, 95% CI:0.68–0.90), the genus RealAmp 99.6% agreement (kappa = 0.98, 95% CI:0.94–0.90) and the genus MG-LAMP 100% agreement (kappa = 1, 95% CI:1–1). Regarding the species-specific assays, the RealAmp showed 94.1% agreement (kappa = 0.85, 95% CI:0.76–0.95) and the MG-LAMP 94.9% agreement (kappa = 0.87, 95% CI:0.78–0.96).

We did not find statistical differences between the results of the RealAmp and the MG-LAMP (McNemar test (p>0.05)).

### Processing time and cost analysis

We estimated the required processing time and costs of microscopy, nested-PCR, RealAmp and MG-LAMP for the specific identification of *Plasmodium* is shown in (Table 3).

**Table 3:** Cost and turnaround time of LAMP assays compared with nested-PCR and Microscopy.

Procedures	^1^Cost (USD) per sample	^2^Turnaround time (hours)	^3^Hands- on work (Minutes)
**Nested-PCR**	$ 9.05	9	80
**RealAmp**	$ 6.52	3	40
**MG-Lamp**	$ 4.73	3	40
**Microscopy**	$0.94	2	40*

^**1**^Estimated cost includes reagents, materials for column-based DNA extraction and PCR and does not include labor costs.

^**2**^Turnaround time is the time required from samples processing to getting results (Includes: DNA extraction, master mix preparation, PCR reaction, and gel electrophoresis (if applicable)

^**3**^Hands-on work is the time spend in the laboratory preparing reactions.

* refers to slide reading time only.

## Discussion

In the context of malaria elimination, the development of simpler and more sensitive parasite detection approaches is a priority [6]. However, most molecular methods such as conventional PCR are not applicable in many resource- limited regions. In this regard, LAMP approaches appear a promising alternative to PCR-based tests given that they are more sensitive than microscopy and RDT’s and are simpler to perform than PCR-based assays [19, 23].

Our study showed the feasibility of utilizing both the RealAmp and MG-LAMP methods for detection of malaria parasites in limited resource settings. Both LAMP assays had similar performance and as expected showed higher sensitivity than microscopy. However, compared to the RealAmp assay, the MG-LAMP appears to be a better choice than the RealAmp assay for the following reasons: 1) the MG-LAMP is less expensive than the RealAmp, 2) MG-LAMP does not require special equipment for readout since it is a colorimetric test and 3) it uses a portable mini heat-block with a capacity of testing up to 36 samples per run which allows for the large scale testing of samples. Preliminary data show that both LAMP assays can be performed using DNA obtained via simpler extraction methods such as the boil and spin method previously reported [16], thus avoiding expensive and time-consuming DNA preparation approaches.

Regarding the limit of detection (LoD), both genus LAMP assays detected up to 2.5 parasites/μL which is comparable to other LAMP assays previously described [12, 16]. The species- specific assays have LoD of 5–10 parasites/μL which is higher than the desired 2 parasites/uL limit. Improvements to these species-specific LAMP assays will be required if the desired LoD is to be achieved; this may be possible with the use of novel primers that can increase the sensitivity of the assays. However, even the current LoDs surpass the diagnostic capacity of the conventional tests, microscopy (50 parasites/μL) and RDT’s (between 200 parasites/μL), suggesting that the LAMP assays may already be useful as alternative parasite detection tests in place of the sophisticated and more expensive conventional nested-PCR for surveillance. Even though the assays showed lower limits of detections, one sample was only detected by genus RealAmp, but not by the species-specific assay. This could be explained due the better performance of genus primers than the species-specific primers [19].

Our results showed that only 9% of mixed infections detected by nested-PCR were detected by microscopy in contrast to 36% by RealAmp and MG-LAMP. Both LAMP assays missed the *P*. *vivax* parasites, but where successful detecting *P*. *falciparum*. As recently reported, the LAMP assays are still lacking their ability to detect mixed infections compared to the nested-PCR [17] [19]. Although the majority of mixed infections have been previously associated with mild cases of malaria, there are occasional reports of severe malaria [24]. Misdiagnosis of mixed infections is especially problematic in the case of missed *P*. *vivax* infection, which requires radical cure of hypnozoites to avoid a relapse. Therefore, efforts should be made to improve sensitivity for mixed infections which is key for appropriate treatment and case management.

A limitation of our study is the fact that all the positive cases were from the symptomatic passive case detection study. Given the low LoD observed for the two LAMP assays one would envision that these assays would be capable of detecting submicroscopic and sub-RDT cases. However, this must be demonstrated in further studies. Surveillance tools for malaria elimination are still required if the elimination goal is to be achieved. Our findings suggest that it is possible to implement simpler molecular tests in facilities with limited resources. However, further improvements are required to increase the assays’ ability to accurately detect all infecting species in cases of mixed infections and the utility of these assays for the detection of asymptomatic cases is required.
